# Classification of true progression after radiotherapy of brain metastasis on MRI using artificial intelligence: a systematic review and meta-analysis

**DOI:** 10.1093/noajnl/vdab080

**Published:** 2021-07-01

**Authors:** Hae Young Kim, Se Jin Cho, Leonard Sunwoo, Sung Hyun Baik, Yun Jung Bae, Byung Se Choi, Cheolkyu Jung, Jae Hyoung Kim

**Affiliations:** Department of Radiology, Seoul National University Bundang Hospital, Seoul National University College of Medicine, Gyeonggi-do, Korea

**Keywords:** artificial intelligence, magnetic resonance imaging, radiosurgery, radiotherapy, systematic review

## Abstract

**Background:**

Classification of true progression from nonprogression (eg, radiation-necrosis) after stereotactic radiotherapy/radiosurgery of brain metastasis is known to be a challenging diagnostic task on conventional magnetic resonance imaging (MRI). The scope and status of research using artificial intelligence (AI) on classifying true progression are yet unknown.

**Methods:**

We performed a systematic literature search of MEDLINE and EMBASE databases to identify studies that investigated the performance of AI-assisted MRI in classifying true progression after stereotactic radiotherapy/radiosurgery of brain metastasis, published before November 11, 2020. Pooled sensitivity and specificity were calculated using bivariate random-effects modeling. Meta-regression was performed for the identification of factors contributing to the heterogeneity among the studies. We assessed the quality of the studies using the Quality Assessment of Diagnostic Accuracy Studies 2 (QUADAS-2) criteria and a modified version of the radiomics quality score (RQS).

**Results:**

Seven studies were included, with a total of 485 patients and 907 tumors. The pooled sensitivity and specificity were 77% (95% CI, 70–83%) and 74% (64–82%), respectively. All 7 studies used radiomics, and none used deep learning. Several covariates including the proportion of lung cancer as the primary site, MR field strength, and radiomics segmentation slice showed a statistically significant association with the heterogeneity. Study quality was overall favorable in terms of the QUADAS-2 criteria, but not in terms of the RQS.

**Conclusion:**

The diagnostic performance of AI-assisted MRI seems yet inadequate to be used reliably in clinical practice. Future studies with improved methodologies and a larger training set are needed.

**Key Points**

- The performance of AI-assisted MRI seems yet inadequate for use in clinical practice.
- All studies used radiomics, and none used deep learning.
- Quality and study design of the published literature should be improved.

**Importance of the Study**

Classification of true progression after stereotactic radiotherapy or radiosurgery of brain metastasis is important, as incorrect diagnosis may lead to unnecessary systemic therapy or additional radiation therapy, or invasive biopsy or surgery for a definitive diagnosis. However, such classification is known to be difficult using advanced imaging modalities such as positron emission tomography or MR spectroscopy, as well as conventional MRI. Our study contributes to the knowledge gap regarding the status of research using artificial intelligence on diagnostic task. Our study reviews the methodology and quality of the current studies, offering valuable information for future research.

Stereotactic radiotherapy or radiosurgery, owing to its high efficacy with relatively short treatment time and favorable toxicity profile, is increasingly used for patients with a limited number of brain metastases.^1^ Contrast-enhanced MR imaging remains the modality of choice for follow-up after stereotactic radiotherapy or radiosurgery (hereinafter, collectively termed as stereotactic radiotherapy) of brain metastasis, as it shows excellent soft-tissue contrast that can delineate structural abnormalities with high resolution. However, new or enlarging lesion on MRI may complicate patient management during follow-up, as such lesion is not always indicative of true progression.^2^ Classification of true progression from nonprogression including radiation necrosis is known to be difficult on conventional MRI. In a previous systematic review,^3^ the pooled sensitivity and specificity of conventional gadolinium MRI across four studies was around 63% and 82%, respectively. Radiation necrosis, which strikingly mimics true progression not only in MR imaging appearance but also in clinical symptoms,^4^ is reported to occur in up to one-fourth of patients after stereotactic radiotherapy.^5^ Incorrect classification of true progression may lead to substantial patient harm, as unnecessary systemic therapy or additional radiation therapy could be administered, or subsequent biopsy or resection may accompany complications such as infection or neurologic deficit. Other advanced imaging modalities such as perfusion MRI, magnetic resonance spectroscopy, 18FLT, 18FDG PET, or SPECT^3^ have also been proposed, but to date, none of those has emerged as a standard for diagnosing true progression.

Artificial intelligence (AI), which is receiving increasing attention as a potential game-changer in the field of medical sciences, may be an alternative solution to the diagnostic challenge at hand. For example, automated quantitative analysis of tumor response for glioblastoma on MRI using artificial neural networks showed reliable performance in an independent dataset for external validation.^6^ However, the scope and status of research using AI on classifying true progression are uncertain at this point. Thus, through this systematic review and meta-analysis, we aimed to measure the diagnostic performance of AI-assisted MRI in classifying true progression from nonprogression after radiotherapy of brain metastasis and to identify factors attributable to the heterogeneity in the included studies.

## Materials and Methods

We adhered to the standard guidelines of Preferred Reporting Items for Systematic Reviews and Meta-Analyses (PRISMA).^7^

### Literature Search

We performed a literature search of the MEDLINE and EMBASE databases using the search terms as follows: ((brain metastas*) OR (cerebral metastas*) OR (metastatic brain tumor) OR (intra-axial metastatic tumor)) AND ((automated) OR (computer aided) OR (computer-aided) OR (CAD) OR (radiomic*) OR (texture analysis) OR (deep learning) OR (machine learning) OR (neural network) OR (artificial intelligence)) AND ((gamma-knife) OR (radiotherapy) OR (radiation) OR (radiosurgery)). The literature search was not restricted to any publication date or study setting, and the search was updated until November 11, 2020. The search was limited to publications in English. Bibliographies of the retrieved studies were manually cross-checked to identify any study meeting the inclusion criteria but were not retrieved using our search terms.

### Inclusion Criteria

Inclusion criteria for the enrollment of studies were as follows: (1) involved patients who received stereotactic radiotherapy for clinically or pathologically diagnosed brain metastasis, (2) used MRI with the aid of AI as the index test (hereinafter, AI-assisted MRI), (3) purposed to show the diagnostic performance of the index test in classifying (ie, either prediction or differentiation) true progression from nonprogression, and (4) provided the information necessary for the reconstruction of 2 × 2 contingency tables. The term “nonprogression” refers collectively to treatment response any other than true progression, including radiation necrosis.

### Exclusion Criteria

The exclusion criteria for the enrollment of studies were as follows: (1) case reports or series including less than ten patients; (2) conference abstracts, editorials, letters, consensus statements, guidelines, or review articles; (3) studies with, or with suspicion of, overlapping populations; (4) study purpose not in the field of interest, which was to estimate diagnostic performance of the AI-assisted MRI in classifying true progression from nonprogression, and (5) insufficient data for the reconstruction of 2 × 2 contingency tables.

Literature search and selection were performed independently by two radiologists (H.Y.K. and S.J.C. with 6 and 7 years of experience in radiology, respectively). Any disagreement between the two reviewers was resolved via consultation of the third reviewer (L.S., with 10 years of experience in neuroradiology, and six years of experience in AI research).

### Data Extraction

Data extraction was performed in a standardized form in adherence to the PRISMA guideline.^7^ We extracted the following data: (1) characteristics of the included studies: authors, year of publication, institution, country of origin, study period, study design (prospective vs retrospective), whether radiomics was used, whether DL was used, patient population from which classification was made (limited to radiation necrosis vs. extended to other conditions of nonprogression including stable disease or regression), method of internal validation, whether external validation was performed, number of included patients, male to female ratio, number of included tumor, proportion of true progression, proportion of lung cancer as the primary site, reference standard, and inclusion and exclusion criteria; (2) characteristics of MRI: machine, field strength, in-plane resolution, slice thickness, dimension, MRI scan point (pre- or postradiotherapy), and sequence used for analysis; (3) characteristics of radiomics (as all studies in the final selection turned out to have used radiomics): segmentation slice (2D [region of interest in two dimension] vs. 3D [volume of interest in three dimension]), subregion segmentation, method of segmentation (manual vs semiautomatic), use of voxel size resampling, filter, normalization, and discretization; (4) characteristics of model development: feature selection method, classification method, number of extracted radiomics feature, and finally selected feature number.

### Quality Assessment

Two reviewers (H.Y.K. and S.J.C.) independently assessed and achieved consensus for the methodological quality of the enrolled studies using the Quality Assessment of Diagnostic Accuracy Studies-2 (QUADAS-2) criteria^8^ and the six domains of the Radiomics Quality Score (RQS) by Park et al.^9,10^ The RQS originally suggested by Lambin et al.^9^ consists of 16 components, with a maximal achievable score of 36. Park et al.^10^ categorized the 16 components of the RQS into 6 domains, where a score of at least 1 point without minus points in each domain was regarded as adherence. The six domains are as shown in Supplementary Table 1. Detailed definitions of each component could be found in Lambin et al.^9^

### Data Synthesis and Analyses

The primary endpoint of the current systematic review and meta-analysis was to measure the diagnostic performance of AI-assisted MRI in classifying true progression from nonprogression. The secondary endpoint was to identify factors attributable to the heterogeneity in the included studies.

We measured the pooled sensitivity and specificity with their 95% confidence intervals (CIs) using bivariate random-effects modeling.^11–15^ We presented the results graphically using hierarchical summary receiver operating characteristic (HSROC) curves with 95% confidence and prediction regions. Publication bias was analyzed using Deeks’ funnel plot, with Deeks’ asymmetry test being used to calculate the *P*-value and determine statistical significance.^16^ Heterogeneity across the selected studies was evaluated using the Cochran Q test, where value *P*-value < .05 indicated the presence of heterogeneity.^17^ According to the Higgins I^2^ statistic, heterogeneity was classified as follows: 0–40%, might not be important; 30–60%, moderate heterogeneity; 50–90%, substantial heterogeneity; and 75–100%, considerable heterogeneity.^12^ The presence of a threshold effect (a positive correlation between sensitivity and false-positive rate) was sequentially evaluated: initially via visual assessment of the coupled forest plots of sensitivity and specificity; and secondarily via Kendall’s Tau, with a *P*-value of less than 0.05 indicating the presence of the threshold.^18^

To determine the factors attributable to heterogeneity across the studies, we performed meta-regression analyses using the following covariates: (1) study characteristics (total tumor number, the multiplicity of tumor per patient, the ratio of true progression to nonprogression, proportion of lung cancer, proportion of pathologically confirmed tumor, patient group), (2) MRI characteristics (MR field strength used, MR sequence used), and (3) radiomics characteristics (number of extracted radiomics feature, delta radiomics, segmentation method, segmentation slice, and voxel size resampling). One of the authors (S.J.C., with three years of experience in performing systematic reviews and meta-analyses) performed the statistical analyses using the MIDAS and METANDI modules in STATA 16.0 (StataCorp).

## Results

### Literature Search

**Figure 1.**
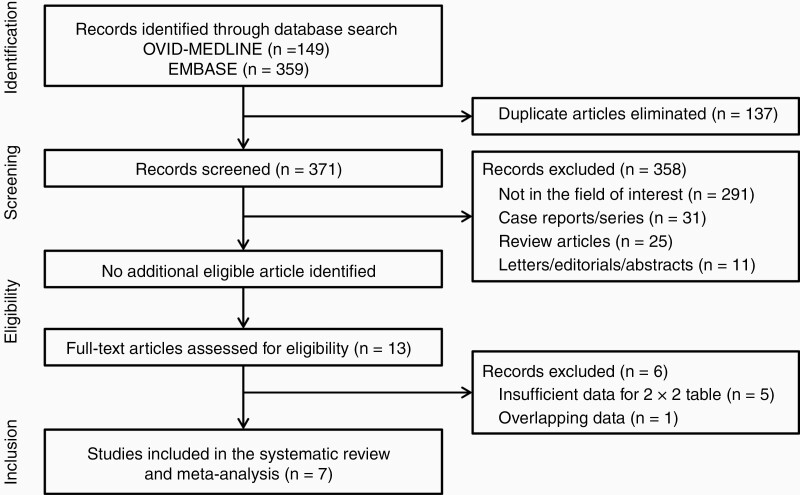
Flow diagram of the study selection process.

Our literature search identified 508 studies initially (Figure 1). After removing 137 duplicates, the remaining 371 studies were screened mainly at the title and abstract level and the full-text level if necessary, yielding 13 potentially eligible studies. No additional study was identified after a manual review of those 13 studies’ bibliographies. After a full-text review of the 13 eligible studies, six studies were excluded for the reasons as follows: five studies had insufficient information for the reconstruction of 2 × 2 table,^19–23^ and one study had overlap in the study population with one of the finally included studies.^24^ Finally, seven studies^24–30^ were included in the present systematic review and meta-analysis.

### Characteristics of the Included Studies

All studies used radiomics with retrospective design to classify true progression from nonprogression on AI-assisted MRI (Table 1). Except for two studies,^24,28^ all studies delimited nonprogression to cases of radiation necrosis. Thus, all studies except for those two studies were of case-control design. All studies lacked external validation of their results. The number of patients across all studies was 485, with the number in individual studies ranging from 20 to 100 patients (Table 1). The number of tumors across all studies was 907, with the number in individual studies ranging from 20 to 408. Five studies^24,26,28-30^ included multiple tumors per patient in the analysis. The proportion of tumors adjudicated to be true progression ranged from 7.8% (32/408) to 75% (73/97) across the studies. The proportion of lung cancer as the primary site ranged from 25% (21/84) to 75% (15/20). The reference standard for true progression and nonprogression was based on pathology and clinical follow up in five studies,^24,26,27,29,30^ on pathology alone in one study,^25^ and clinical follow up alone in one another study.^28^ The details of the inclusion and exclusion criteria in each study were described in Table 1.

**Table 1.** Characteristics of the Included Studies

Source	Affiliation	Study period	Study design	Classification of true progression	Validation		Patient		Tumor			Reference standard	Criteria	
					Intern.	Extern.	Total no.	M/F ratio	Total tumor no.	Proportion of true progression*	Proportion of lung cancer†		Inclusion	Exclusion
Hettal 2020	Lorraine Comprehensive Cancer Center, France	2008–2017	Retro	From radiation necrosis	Leave one out CV	No	20	10:10	20	60% [12/20]	75% [15/20]	Pathology	New or enlarging contrast-enhancing lesion after SRT; Adult; New oligometastasis‡; KPS 70% or higher	Diagnosis of radiation necrosis obtained after re-irradiation
Karami 2019	Sunnybrook Health Sciences Centre (SHSC), Canada	NA	Retro	Yes	Leave one out CV	No	100	37:63	133ǁ	40% [53/133]	49% [65/133]	Pathology and clinical (RANO-BM) follow-up	Metastasis and treated with SRT	NA
Larroza 2015	Universitat de Vale` ncia, Spain	September 2007-June 2013	Retro	From radiation necrosis	Internal split¶	No	73	37:36	115ǁ	72% [83/115]	NA	Pathology and clinical (RECIST) follow-up	New or enlarging contrast-enhancing lesion after SRT; Pathologically proven primary extra-cerebral tumor	Nonparenchymal metastasis; SRS performed for consolidation to a surgical cavity
Lohmann 2018	Forschungszentrum Juelich, Inst. of Neuroscience and Medicine, Germany	2006–2014	Retro	From radiation necrosis	Leave one out, 5-fold and 10-fold CV	No	52	13:39	52	40% [21/52]	52% [27/52]	Pathology and clinical (RANO-BM) follow-up	New or enlarging contrast-enhancing lesion after SRT	Lack of information regarding positron emission tomography
Mouraviev 2020	University of Toronto, Canada	December 2016-November 2017	Retro	From nonprogression§	Leave one out CV	No	87	35:52	408ǁ	7.8% [32/408]	49.5% [202/408]	Clinical (RANO-BM) follow-up	Contrast-enhancing metastasis; Pathologically proven primary extra-cerebral tumor	Nonparenchymal or cystic metastasis; Surgical cavities; Received previous SRS
Peng 2018	Johns Hopkins University School of Medicine, USA	June 2003–September 2017	Retro	From radiation necrosis	10-fold CV**	No	66	NA	82ǁ	63% [52/82]	34% [28/82]	Pathology and clinical follow-up††,‡‡	New or enlarging contrast-enhancing lesion after SRT	Poor MRI quality
Zhang 2018	University of Texas MD Anderson Cancer Center, USA	August 2009-August 2016	Retro	From radiation necrosis	Leave one out CV	No	87	46:38§§	97ǁ	75% [73/97]	25% [21/84]§§	Pathology and clinical follow-up‡‡	New or enlarging contrast-enhancing lesion after SRT; At least two MR scans obtained after SRT but before confirmation	Poor MRI quality

DL: deep learning, Int.: internal, Ext.: external, no.: number, M/F: Male/Female, Retro.: retrospective, CV: cross validation, SRT(S): stereotactic radiotherapy (surgery), KPS: Karnofsky performance score, RANO-BM: Response assessment in neuro-oncology brain metastases, NA: not available, RECIST: response evaluation criteria in solid tumors, MRI: magnetic resonance imaging.

*Out of all tumors;

^†^Out of all tumors, except for Zhang 2018 in which the patient number was used as the denominator;

^‡^Fewer than five, measuring 5 mm or more but not exceeding 4 cm;

^§^Nonprogression including radiation necrosis. These two studies were regarded as cohort studies in the meta-regression;

^ǁ^Inclusion of multiple tumors per-patient;

^¶^Merged training and test sets and repeated internal split 100 times holding 70% in training, and then averaged AUC values of test sets;

^**^100 iterations;

^††^All true progression cases were proven by pathology;

^‡‡^Information regarding the reference standard for clinical follow-up was unavailable;

^§§^Out of 84 patients included in the original table.

### MRI, Radiomics, and Model Development in the Included Studies

Information regarding MR field strength, in plane resolution, and slice thickness is detailed in Table 2. Except for one study^28^ that only used images acquired before radiotherapy, all studies used images acquired after radiotherapy. One study^24^ used images acquired both before and after radiotherapy. Contrast-enhanced T1 weighted images were used for the analysis in all studies, while T2 FLAIR or T2 weighted images were analyzed additionally in five studies.^24,26,28–30^ Many studies lacked detailed information regarding image segmentation for radiomics feature extraction. The region used for radiomics feature extraction in each study is summarized in Supplementary Table E2. Image segmentation was conducted semi-automatically in five studies^24,26,28–30^ and manually in the remaining two studies.^25,27^ There was variable use of the radiomics techniques; voxel size resampling was used in four studies,^24,25,27,28^ filtering in four studies,^24,27,28,30^ image normalization in four studies,^25–28^ and discretization in four studies.^25–27,29^ The categories of radiomics features used in each study are summarized in Supplementary Table E3. The number of extracted radiomics features ranged from 42 to 3072 across the studies, with more than 400 features used in three studies,^24,25,28^ and less than 400 features used in the remaining four studies.^26,27,29,30^ Finally selected feature numbers ranged from four to 12. Detailed feature selection methods and classification methods are summarized in Table 2.

**Table 2.** Characteristics of MRI, Radiomics, and Model Development

Source	MRI							Radiomics										
								Segmentation			Technique				Model			
	Machine	T	In-plane resolution (mm)*	Slice thickness (mm)*	D	Scan Point	Sequence used for analysis	ROI vs. VOI	Subregion segmentation	Method	Voxel size resampling	Filter	Normalization	Discretization	Feature selection method	Classification method	NO. of extracted radiomics features†	Finally selected feature number
Hettal 2020	NA	1.5 or 3	NA	NA	3D	Post SRT	T1W C+	VOI	Not used	Manual	Used	Not used	Used	Used	Univariate analysis (filter approach)	Bagging algorithm	1766	4
Karami 2019	Ingenia, Philips	1.5	0.5	1.5	NA	Both pre and post SRT‡	T1W C+, T2 FLAIR	VOI	Used§	Semiautomatic	Used	Used	Not used	Not used	Pearson correlation analysis, Mann-Whitney U test	SVM classifier with bootstrap	3072	5
Larroza 2015	Magnetom Symphony, Siemens	1.5	0.5	1.3	3D	Post SRT	T1W C+, T2 FLAIR	ROI	Not used	Semiautomatic	Not used	Not used	Used	Used	Mann-Whitney U test with Benjamini-Hochberg correction	SVM classifier with recursive feature elimination	179	7
Lohmann 2018	NA	NA	NA	NA	NA	Post SRT	T1W C+	VOI	Not used	Manual	Used	Used	Used	Used	Mann-Whitney U test	Generalized linear model by applying AIC	42	5
Mouraviev 2020	Ingenia, Philips	1.5	NA	NA	3D	Pre SRT	T1W C+, T2 FLAIR	NA	Usedǁ	Semiautomatic	Used	Used	Used	Not used	Resampled random forest feature importance	Random forest classifier	440	12¶
Peng 2018	Philips, Siemens, General Electric	1.5 or 3	0.43–1.02	0.9–5	NA	Post SRT	T1W C+, T2 FLAIR	ROI	Not used	Semiautomatic	Not used	Not used	Not used	Used	Univariate logistic regression performance (AUC)	SVM classifier	51	5
Zhang 2018	Signa HDXt, General Electric	1.5	NA	5	NA	Post SRT‡	T1W, T1W C+, T2W, FLAIR	VOI	Not used	Semiautomatic	Not used	Used	Not used	Not used	Concordance correlation coefficients	Ensemble classifier	285	5

MRI: magnetic resonance imaging, T: tesla, field strength, D: dimension, ROI: region of interest, VOI: volume of interest, NO.: number, NA: not available, 3D: 3 dimensional, SRT: stereotactic radiotherapy or radiosurgery, T1W C+: T1 weighted contrast-enhanced, FLAIR: fluid attenuated inversion recovery, SVM: support vector machine, AIC: Akaike Information Criterion, AUC: area under receiver operating characteristics curve.

*For T1W C+ images;

^†^For each ROI or VOI;

^‡^Delta radiomics;

^§^(1) Enhancing region in T1W images (tumor), (2) Edema, (3) Isotropic expansion around the tumor and edema, (4) Isotropic expansion around the tumor;

^ǁ^Tumor core and the peritumoral regions;

^¶^Including 3 clinical features.

### Diagnostic Performance of the MRI

**Figure 2.**
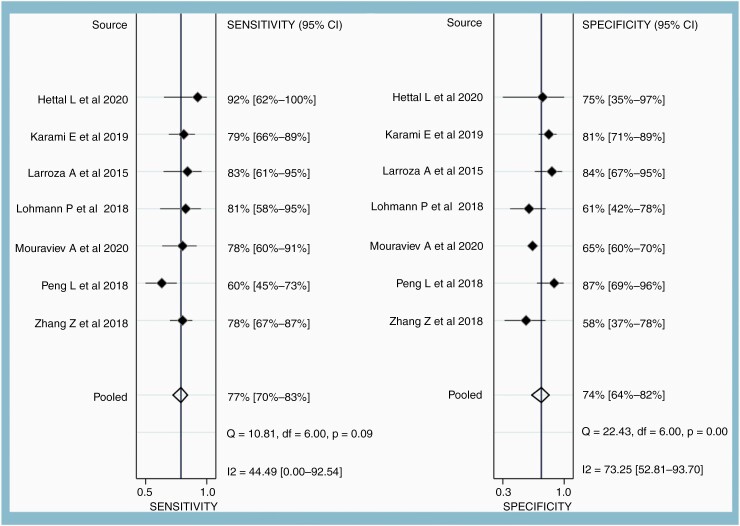
Forest plots showing pooled sensitivity and specificity of AI-assisted MRI in classifying true progression from nonprogression after stereotactic radiotherapy of brain metastasis. Horizontal error bars and black diamonds represent 95% confidence intervals and point estimates of each study, respectively. Solid vertical lines represent pooled point estimates.

**Figure 3.**
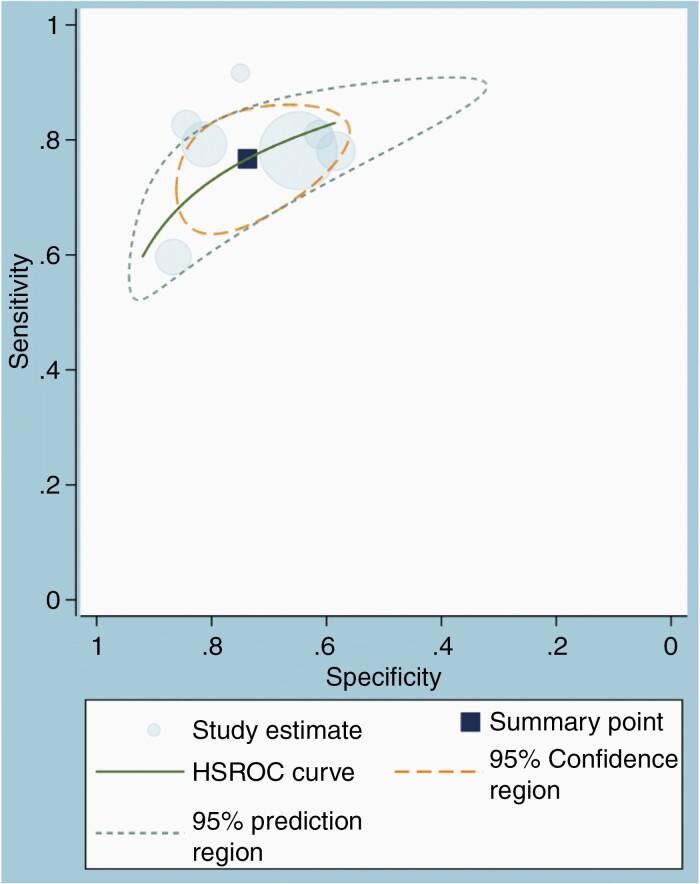
Hierarchical summary receiver operating characteristic (HSROC) curve showing the performance of AI-assisted MRI.

Across the seven studies, the pooled sensitivity was 77% (95% CI, 70–83%), and the pooled specificity was 74% (95% CI, 64–82%). The range of sensitivity and specificity across the seven studies was 60–92% and 58–87%, respectively (Figure 2). The area under the HSROC curve was 0.82 (95% CI, 0.78–0.85) (Figure 3). The difference between the 95% confidence and the prediction regions was relatively large, indicating heterogeneity among the studies. According to the Q test, heterogeneity was present (*P* = .026), mainly due to the heterogeneity in the specificity (*P* < .01) and not sensitivity (*P* = .09). Higgins I^2^ statistics were also suggestive of heterogeneity that “might not be important” in the sensitivity (I^2^ = 44.5%) and moderate heterogeneity in the specificity (I^2^ = 73%). There was no threshold effect (Kendall’s Tau value of −0.04, *P* = .76). According to Deeks’ funnel plot, the likelihood of publication bias was low, with a *P*-value of .54 for the slope coefficient (Supplementary Figure 1).

### Meta-regression

In the meta-regression analysis (Table 3), several covariates showed a statistically significant association with the heterogeneity in the joint model. Those factors were the proportion of lung cancer as the primary site, proportion of pathologically confirmed tumor, MR field strength used, and segmentation slice. Sensitivity was increased while specificity was lowered, in the studies with 50% or higher proportion of lung cancer as the primary site, with less than 50% of the pathologically confirmed tumor, and in the studies that used MR field strength of 1.5T only, and VOI in segmentation.

**Table 3.** Meta-Regression of MRI Radiomics for Classifying True Progression from Nonprogression

Covariate	Subgroup	Meta–analytic summary estimate		*P*-value
		Sensitivity [95% CI]	Specificity [95% CI]	
**Study characteristics**				
Total tumor number	<100	80% [70%–90%]	73% [58%–87%]	.78
	≥100	75% [67%–83%]	74% [63%–85%]	.78
Multiplicity of tumor per patient	No	85% [72%–98%]	65% [44%–86%]	.40
	Yes	75% [68%–82%]	76% [67%–85%]	.40
Ratio of true progression to nonprogression	≤1.5	80% [72%–89%]	70% [58%–82%]	.51
	>1.5	74% [65%–82%]	77% [66%–89%]	.51
Proportion of lung cancer*	<50%	74% [67%–82%]	74% [64%–84%]	<.001
	≥50%	85% [72%–98%]	65% [44%–86%]	<.001
Proportion of pathologically confirmed tumor†	<50%	80% [70%–90%]	69% [56%–82%]	<.001
	≥50%	73% [63%–83%]	74% [59%–89%]	<.001
Patient group	Cohort	80% [70%–90%]	73% [58%–87%]	.78
	Case control	75% [67%–83%]	74% [63%–85%]	.78
**MRI**				
MR field strength used	1.5 Tesla only	79% [73%–85%]	73% [63%–82%]	<.001
	3 Tesla	66% [54%–77%]	84% [70%–98%]	<.001
MR sequence used	T1W C+ only	85% [72%–98%]	65% [44%–86%]	.40
	Others also	75% [68%–82%]	76% [67%–85%]	.40
**Radiomics**				
Number of extracted radiomics feature‡	<400	74% [65%–82%]	74% [62%–86%]	.45
	≥400	81% [72%–90%]	72% [59%–85%]	.45
Delta radiomics	Not used	75% [66%–84%]	74% [63%–84%]	.86
	Used	78% [69%–88%]	74% [58%–89%]	.86
Segmentation Method	Manual	85% [72%–98%]	65% [44%–86%]	.40
	Semiautomatic	75% [68%–82%]	76% [67%–85%]	.40
Segmentation slice	VOI	80% [74%–86%]	71% [61%–81%]	<.001
	ROI	67% [56%–77%]	86% [76%–96%]	
Voxel size resampling	Not used	72% [63%–81%]	78% [66%–90%]	.27
	Used	81% [74%–89%]	70% [59%–81%]	.27

tCI: confidence interval, T1 W C+: T1 weighted contrast-enhanced, VOI: volume of interest, ROI: region of interest.

*Out of all tumors, except for in Zhang et.al in which the patient number was used as the denominator;

^†^Out of all tumors;

^‡^Per region- or volume of interest.

### Quality Assessment

**Figure 4.**
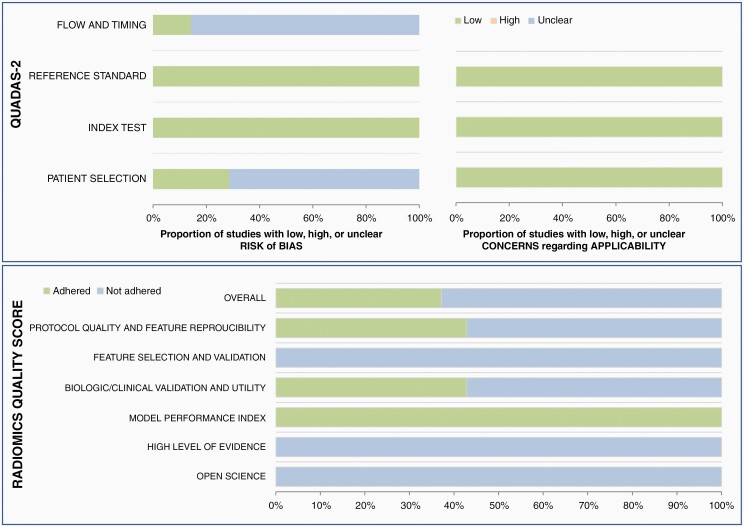
Quality assessment of the studies using the Quality Assessment of Diagnostic Accuracy Studies-2 (QUADAS-2) and the radiomics quality score (RQS). In the flow and diming domain, six studies were considered to have an unclear risk of bias, since not all patients underwent the same reference standard procedure but were adjudicated based on either pathology or clinical follow-up results.

Overall ratings were favorable in terms of the QUADAS-2 criteria (Figure 4). In the patient selection domain, 5 studies were considered to have an unclear risk of bias due to the case-control study design and unclear information regarding inappropriate exclusion.^25–27,29,30^ In the flow and timing domain, 6 studies were considered to have an unclear risk of bias, since not all patients underwent the same reference standard procedure, but were adjudicated based on either pathology or clinical follow-up results. Otherwise, the bias risks in the index test and reference standard were regarded as low in all studies. There was low concern regarding applicability in the patient selection, index test, and the reference standard for all studies.

The quality of the studies was further assessed using RQS. The scores were low (below 4) in all studies. All studies^24–30^ showed adherence to the model performance index (domain 4). However, only three studies showed adherence to domain 1 (protocol quality and stability in image and segmentation),^26,29,30^ and another three studies to domain 3 (biologic/clinical validation and utility).^25,28,29^ Furthermore, none of the studies adhered to domain 2 (feature selection and validation), domain 5 (high level of evidence), and domain 6 (open science and data). The detailed score according to the domains was presented in Supplementary Table 1.

## Discussion

This systematic review and meta-analysis included seven studies that aimed to classify true progression after stereotactic radiotherapy of brain metastasis on MRI with the aid of AI. Across the seven studies^24–30^ including 485 patients and 907 tumors, the pooled sensitivity and specificity were 77% (95% CI, 70–83%) and 74% (64–82%), respectively. Heterogeneity was present, mainly in the specificity but not sensitivity. Study quality was overall favorable in terms of the QUADAS-2 criteria, but not in terms of the RQS.

As a classification of true progression on standard MRI alone is difficult, other advanced imaging modalities such as MR perfusion, MRS, or PET have also been proposed.^31–34^ Although the pooled sensitivities and specificities across the studies that investigated those advanced imaging modalities were generally above 80% according to a previous systematic review,^3^ the Response Assessment in Neuro-Oncology Brain Metastases (RANO-BM) working group considers those previous studies as inadequately robust to render any solid evidence and thus recommends multidisciplinary team decision rather than relying on any one of those imaging modalities.^35^ In fact, the previous studies included small numbers of patients and lacked external validation. Moreover, due to difficulties in establishing a definitive diagnosis, many of the previous studies were conducted with a case-control design (ie, including only the patients who underwent pathological confirmation), rather than with a cohort including all patients presenting with the new or enlarging enhancing lesion.

Meanwhile, AI has been increasingly utilized in medical imaging, such as for diagnosis and prediction of risk and prognosis. If diagnostic accuracy in classifying true progression after radiotherapy on standard imaging could be improved by using AI, it may usher in a breakthrough in the challenge. However, our study results suggest otherwise, with the performance of AI-assisted MRI not much superior to the reported performances of imaging modalities without the assistance of AI. The disappointing results may be attributable to the inadequate size of training data, inappropriate AI algorithm, or the intricate nature of the challenge that is unsolvable even by applying AI. Robustly designed future studies that address those issues are needed, preferably with a larger number of patients in the training set. Future studies that apply deep learning are also warranted; although our systematic search was targeted for any kind of AI, all retrieved studies had used radiomics. Another way of improving the diagnostic accuracy would be to take temporal changes of imaging findings into account, rather than using data from a single time point (eg, when a new or enlarging enhancing lesion was initially detected on MRI). Although two of our studies^24,30^ had already incorporated such a concept by using delta radiomics and did not show significant improvement in the performance, further research using data from multiple time points (eg, pre-RT, two post-RT images) could be attempted.

Although there was no significant threshold effect, substantial heterogeneity still existed, especially in specificity but not in sensitivity. Several covariates, including the proportion of lung cancer as the primary site, proportion of pathologically confirmed tumor, MR field strength, and segmentation slice, showed a statistically significant association with the heterogeneity. Lung cancer is the most common primary cancer of brain metastasis,^36^ and thus studies with the proportion of lung cancer as the primary site of 50% or higher would better represent the real population compared to those with the proportion lower than 50%. Radiomics feature selection and subsequent analysis are known to be affected substantially by imaging acquisition parameters and reconstruction techniques.^37,38^ Thus, the MR field strength and the segmentation slice used (ROI vs VOI) may have contributed to the heterogeneity in our results. Moreover, in case ROI was used, there is a possibility that the slice selected for feature extraction may not have represented the overall tumor nature appropriately, as the target lesion may be a mixture of both recurrent tumor and radiation necrosis.^39^

Although the quality assessment in terms of the QUADAS-2^8^ was relatively favorable, that in terms of the RQS^9,10^ was generally poor. Adherence was especially low in domain 2 (feature selection and validation), domain 5 (high level of evidence), and domain 6 (open science and data), mostly due to the lack of external validation, prospective study design, and open-source data. Low adherence in domain 6 calls for further efforts in inter-institutional data and model sharing, which is critical in generating reproducible study results. Adherence in domain 1 was also suboptimal in most studies, raising concern regarding the repeatability and reproducibility of the study procedure. Although expectations for AI to be a panacea for our diagnostic challenges are high, there are also concerns that complexity and “black box” nature inherent to AI make it difficult for others to apply the algorithm to clinical workflow or to perform external validation.^40^ Such lack of transparency calls for firm adherence to standardized methodological and reporting procedures. However, there are yet established guidelines for the reporting and quality assessment of the diagnostic accuracy or prognostic studies using AI, which is a relatively nascent methodology. The release of AI-specific extension to the STARD (Standards for Reporting of Diagnostic Accuracy Studies) and TRIPOD (Transparent Reporting of a Multivariable Prediction Model for Individual Prognosis or Diagnosis) is underway,^40,41^ and future studies on classifying true progression after radiotherapy would hopefully be conducted according to those new guidelines.

There were limitations in our study. First, the numbers of studies in each subgroup in the meta-regression were mostly small, possibly inadequate for drawing statistically robust conclusions. Second, there were substantial differences in the methodology across the studies, raising concern in pooling the results. For example, unlike the rest of the studies that reported the diagnostic performance by using radiomics features alone, the study by Mouraviev et al.^28^ had reported the performance of radiomics features in addition to clinical features. Karami et al.^42^ and Zhang et al.^30^ incorporated delta radiomics by using MR images from more than one time point, and Mouraviev et al.^28^ used pre-RT MR images, whereas the remaining studies used only the MR images at a single time point after RT. Moreover, the inclusion of patients who had received whole-brain radiotherapy varied across the studies, with most studies lacking detailed information regarding the patients’ previous treatment history. Nevertheless, we chose to use broad inclusion criteria, and instead analyzed various factors and clinical settings attributable to the heterogeneity affecting the diagnostic performance. Third, only two studies^25,29^ reported the performance of neuro-radiologists on standard MRI without the aid of AI. Thus, it was not possible to measure the added value of the AI compared to the conventional MRI. Fourth, not all step-by-step procedures of radiomics were detailed in the included studies. Thus, substantial heterogeneity caused by varied methodologies across the studies may not have been captured adequately in this systematic review. Nevertheless, all included studies have shared the general pipeline of radiomics (ie, beginning from image acquisition, segmentation, preprocessing, feature extraction, feature selection, to validation of model performance). Methodological heterogeneity in studies using AI, including but not limited to those using radiomics, is almost inevitable. However, we may hopefully have a better understanding of the source of heterogeneity via the research community’s more dedicated data and model sharing.

In conclusion, our systematic review of studies that used AI in classifying true progression after stereotactic radiotherapy of brain metastasis has identified seven studies, all of which had used radiomics but not deep learning. The diagnostic performance of AI-assisted MRI seems yet inadequate to be used reliably in clinical practice. Further studies with improved methodologies and a larger training set are needed.

## Supplementary Material

vdab080_suppl_Supplementary_Materials

## Notes

**Authorship Statement**. H.Y.K: the implementation, and interpretation of the data, writing of the draft manuscript, and approval of the final version. S.J.C.: experimental design, analysis, and interpretation of the data, writing of the draft manuscript, and approval of the final version. L.S.: experimental design, the implementation, and interpretation of the data, writing of the manuscript at the revision stage, and approval of the final version. S.H.B., Y.J.B., B.S.C., C.J., and J.H.K.: analysis and interpretation of the data, writing of the manuscript at the revision stage, and approval of the final version.
