# Supplementary material for: Classification of true progression after radiotherapy of brain metastasis on MRI using artificial intelligence: a systematic review and meta-analysis
Source: Neurooncol Adv. 2021 Jul 1;3(1):vdab080. doi: 10.1093/noajnl/vdab080 (PMC8350153; doi:10.1093/noajnl/vdab080)
Supplement: vdab080_suppl_Supplementary_Materials [file vdab080_suppl_supplementary_materials.docx]

**Supplementary Table 1. Radiomics Quality Score**

| Source | **Domain 1: Protocol quality and stability in image and segmentation** | | | |  | **Domain 2: Feature selection and validation** | |  | **Domain 3: Biologic/clinical validation and utility** | | | |  | **Domain 4: Model performance index** | | |  | **Domain 5: High level of evidence** | | **Domain 6: Open science and data** | |
| --- | --- | --- | --- | --- | --- | --- | --- | --- | --- | --- | --- | --- | --- | --- | --- | --- | --- | --- | --- | --- | --- |
| Source | Protocol quality | Multiple segmentation | Phantom study | Test-retest |  | Feature reduction, adjustment for multiple testing | Validation |  | Multivariable analysis with non radiomics features | Biological correlates | Comparison with gold standard^*^ | Potential clinical utility |  | Discrimination statistics | Calibration statistics | Cut-off analyses |  | Prospective study | Cost effectiveness analysis | Open science and data |  |
| Hettal  2020 | 0 | 0 | 0 | 0 |  | 3 | -5 |  | 0 | 0 | 1 | 0 |  | 2 | 0 | 0 |  | 0 | 0 | 0 |  |
| Karami 2019 | 0 | 0 | 0 | 0 |  | 3 | -5 |  | 0 | 0 | 0 | 0 |  | 2 | 0 | 0 |  | 0 | 0 | 0 |  |
| Larroza 2015 | 1 | 0 | 0 | 0 |  | 3 | -5 |  | 0 | 0 | 0 | 0 |  | 2 | 0 | 0 |  | 0 | 0 | 0 |  |
| Lohmann 2018 | 0 | 0 | 0 | 0 |  | 3 | -5 |  | 0 | 0 | 0 | 0 |  | 2 | 0 | 0 |  | 0 | 0 | 0 |  |
| Mouraviev 2020 | 0 | 0 | 0 | 0 |  | 3 | -5 |  | 1 | 0 | 0 | 0 |  | 2 | 0 | 0 |  | 0 | 0 | 0 |  |
| Peng  2018 | 0 | 1 | 0 | 0 |  | 3 | -5 |  | 1 | 0 | 1 | 0 |  | 2 | 0 | 0 |  | 0 | 0 | 0 |  |
| Zhang  2018 | 0 | 0 | 0 | 1 |  | 3 | -5 |  | 0 | 0 | 0 | 0 |  | 2 | 0 | 0 |  | 0 | 0 | 0 |  |

Data are scores of the Radiomics Quality Score (RQS).^1^ Domains of the RQS are adopted from.^2^

^*^Comparison to performance of neuroradiologists.

**Supplementary Table 2. Region of Radiomics Feature Extraction**

| Source | Region of Radiomics Feature Extraction |
| --- | --- |
| Hettal 2020 | Not specified |
| Karami 2019 | 1) Enhancing region in T1W images (tumor), 2) Edema, 3) Isotropic expansion around the tumor and edema, 4) Isotropic expansion around the tumor |
| Larroza 2015 | Most solid lesion component |
| Lohmann 2018 | Not specified. If there were multiple lesions, the lesion with the largest volume was used for segmentation. |
| Mouraviev 2020 | Tumor core and the peritumoral regions |
| Peng 2018 | Not specified. Axial slice showing the 'largest-diameter' was used. |
| Zhang 2018 | Not specified |

**Supplementary Table 3. Region of Radiomics Feature Extraction**

| Source | Categories of Radiomics Features |
| --- | --- |
| Hettal 2020 | Tumor intensity, shape, texture and gray-level features |
| Karami 2019 | Geometrical features, histogram features, local binary patterns, GLCM, wavelet |
| Larroza 2015 | Histogram, absolute gradient, GLCM, GLRLM, autoregressive model, wavelets |
| Lohmann 2018 | Statistical indices, histogram features, GLCM, neighborhood grey-level different matrix, GLRLM, grey-level zone length matrix, and two shape indices |
| Mouraviev 2020 | First order, shape based, GLCM, GLRLM, gray level size zone matrix, neighboring gray tone difference matrix, gray level dependence matrix |
| Peng 2018 | First-order statistics, GLCM, GLRLM, neighborhood gray tone difference matrix, morphologic features |
| Zhang 2018 | Direct intensity and intensity histogram, GLCM, GLRLM, geometric shape, neighborhood gray tone difference matrix, histogram of oriented gradients. |

GLCM = gray level co-occurrence matrix; GLRLM = gray level run length matrix.

**Supplementary Figure 1. Deeks’ Funnel Plot for Evaluation of Publication Bias.**


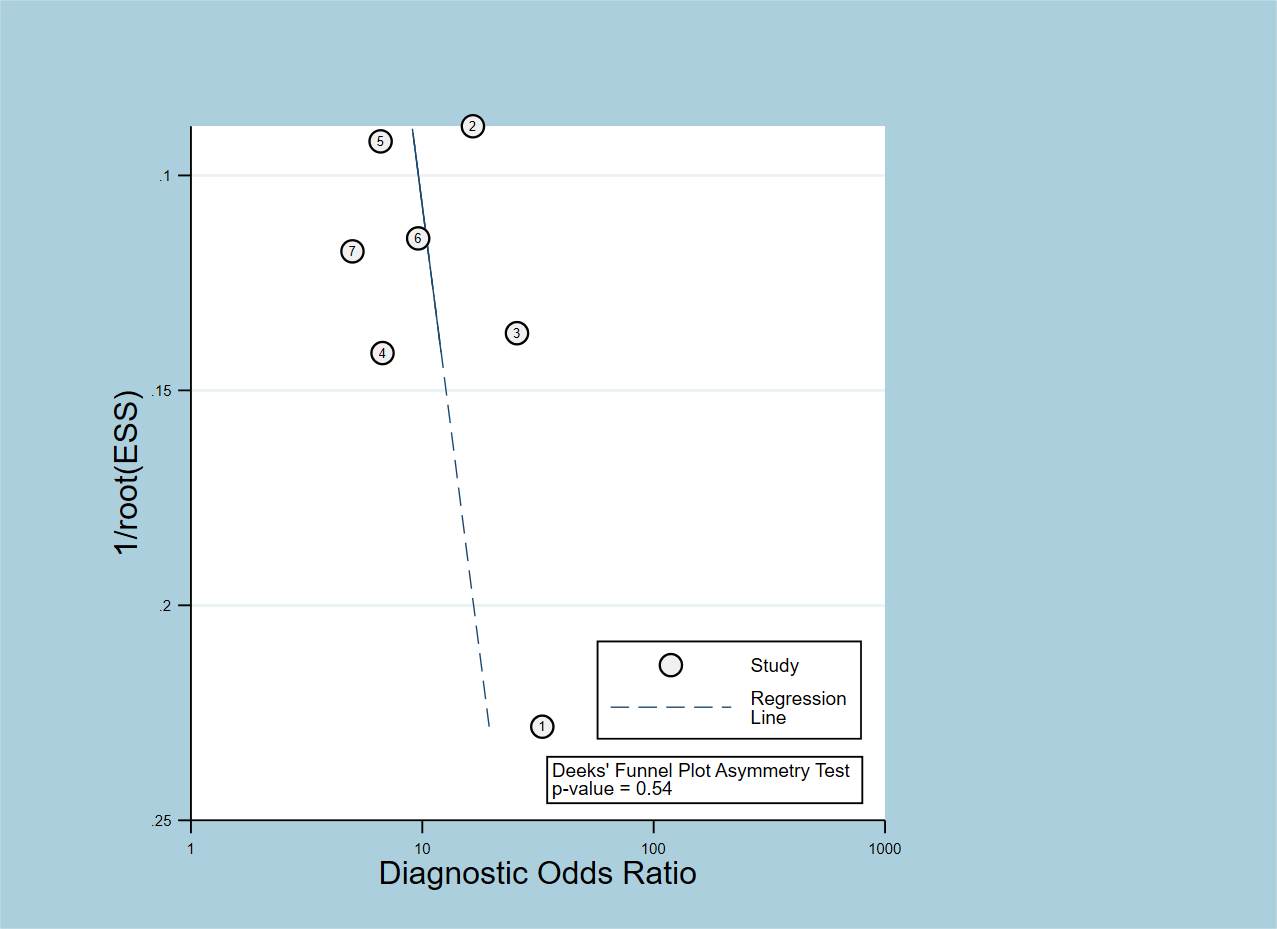


**Reference**

1. Lambin P, Leijenaar RTH, Deist TM et al. Radiomics: the bridge between medical imaging and personalized medicine. *Nat Rev Clin Oncol.* 2017;14(12):749-762.

2. Park JE, Kim HS, Kim D et al. A systematic review reporting quality of radiomics research in neuro-oncology: toward clinical utility and quality improvement using high-dimensional imaging features. *BMC Cancer.* 2020;20(1):29
